# Meeting Report: The 2^nd^ Annual Argonne Soils Workshop, Argonne National Laboratory, Chicago Illinois, USA, October 6-8, 2010

**DOI:** 10.4056/sigs.1874546

**Published:** 2011-11-15

**Authors:** Sarah L. O’Brien, Elizabeth M. Glass, Jennifer M. Brulc, Jack A. Gilbert, Dionysios A. Antonopoulos, Folker Meyer

**Affiliations:** 1 Institute for Genomics and Systems Biology, Argonne National Laboratory, Argonne, IL USA; 2 Mathematics and Computer Science Division, Argonne National Laboratory, Argonne, IL USA; 3 Biosciences Division, Argonne National Laboratory, Argonne, IL USA; 4 Department of Ecology and Evolution, University of Chicago, Chicago, IL USA; 5 Computation Institute, University of Chicago, Chicago, IL USA

## Abstract

This report summarizes the proceedings of the 2^nd^ Annual Argonne Soils Workshop held at Argonne National Laboratory October 6–8, 2010. The workshop assembled a diverse group of soil ecologists, microbiologists, molecular biologists, and computational scientists to discuss the challenges and opportunities related to implementation of metagenomics approaches in soil microbial ecology. The overarching theme of the workshop was “designing ecologically meaningful soil metagenomics research”, which encouraged presentations on both ecological and computational topics. The workshop fostered valuable cross-discipline communication and delivered the message that soil metagenomics research must be based on an iterative process between biological inquiry and bioinformatics tools.

## Introduction

The 2^nd^ Annual Argonne Soils Workshop, held at Argonne National Laboratory October 6–8, 2010 [1], showcased the rapidly maturing subfield of soil metagenomics; over 160 attendees engaged in the 32 scientific talks and 42 posters during the workshop. Soils are, without doubt, the most diverse systems on Earth, making it daunting to characterize their complex, yet ecologically critical microbial communities and to link information about these communities to other important environmental parameters. Continuing advances in molecular biology and bioinformatics have set the stage for a revolution in soil microbial ecology by supplying the detailed data necessary to characterize microbial taxonomy and function. Such prodigious data generation is exciting but presents a real challenge for analysis and interpretation of experimental results. To meet this challenge head on, the overarching theme of the workshop was to discuss how to design experiments and analytical tools capable of distilling meaningful information from the *data deluge*.

The barriers to understanding soil microbial communities are both technical and conceptual, and better integration of theory and research approaches clearly is needed. For instance, few metagenomics datasets from soil are accompanied by adequate data describing the environment from which the physical samples were extracted-- such data could provide critical context for explaining patterns in microbial assemblages. As this develops, it is vital that the those employing metagenomics approaches to soil learn from early adopters in related fields (e.g., marine metagenomics) by implementing appropriate metadata acquisition and databasing protocols so that all datasets will be equally valuable and viable for comparative analysis and provide ecologically meaningful information.

To this end, the 2^nd^ Argonne Metagenomic Workshop built on the success of the 1^st^ workshop held in 2009. The first meeting initiated a dialogue among computation experts and those at the forefront of metagenomics research to build capabilities for visualizing large datasets. The second workshop expanded that dialogue by including more soil ecologists. A broad range of presentations focused on developing better sampling and analytical techniques, exploring biases in the environmental analysis, and improving our fundamental understanding of the ecology and function of soil microbial ecosystems. The result was a dynamic discussion of how to execute ecologically meaningful soil metagenomics research.

## Presentations

### Characterizing the impacts of soil spatial structure on microbial habitats

A primary goal of soil metagenomics is to link microbial community structure and function to essential ecosystem processes. The keynote presentation of the workshop by Josh Schimel (University of California, Santa Barbara) kicked off the workshop with the salient assertion that effective soil metagenomics work has to respect the complexity of the soil environment at spatial and temporal scales that are most relevant to the research question under investigation. Efforts to characterize two-dimensional spatial structuring of microbial communities were presented in several talks and posters, and some even attempted to explore the enormous small-scale heterogeneity of soil aggregates that generates a wide range of habitats for microbes [2] and could be responsible for microbial diversity within a soil. For example, Allan Konopka (Pacific Northwest National Laboratory), Sheri Simmons (Marine Biological Laboratory) and Jennifer Moore-Kucera (Texas Tech University) presented intriguing preliminary data on microbial diversity within soil aggregates. Spatial heterogeneity that impacts microbial activity can also arise from gradients with soil depth, as shown by the work of Janet Jansson (Lawrence Berkeley National Laboratory) and Petr Baldrien (Institute of Microbiology of the ASCR). Konopka also urged participants to consider ways to overcome the substantial challenges associated with measuring temporal patterns in microbial activity since such patterns are vital for understanding the dynamic nature of ecosystem function.

### Parameterizing microbial function

Many of the workshop participants approached the daunting functional complexity of soil by focusing on a particular process, thereby simplifying their experimental inquiry and gaining insight to ecological function. Jay Lennon (Kellogg Biological Station at Michigan State University) demonstrated the disproportionately important role of rare taxa in response to moisture pulses and explained how this could maintain the extraordinary diversity observed in soil microbial communities [3,4]. Likewise, Cheryl Kuske (Los Alamos National Laboratory) explored how changes in the soil environment induced by elevated atmospheric CO_2_ affect microbial communities. Tom Schmidt (Michigan State University) showed how concentrating on genes specific to the soil nitrogen cycle can reveal mechanisms responsible for emission of critical greenhouse gases. Other examples included Juan Imperial’s (UMP – INIA & C.S.I.C, Spain) work on rhizobia mutualisms with plant roots and Stefan Green’s (Florida State University) research on denitrification in a nitrate-contaminated site. Such focus can also be applied to the bioinformatics component of metagenomics research, as Lee Taylor (University of Alaska) demonstrated with his work to modify bacteria-centric informatics tools for the unique requirements of fungal data. Several speakers noted that gene-targeted metagenomics, while still in its infancy, is a promising approach to soil-centric questions.

The power of manipulative experiments, especially companion field and lab experiments, also surfaced as an approach with substantial utility. Examples included Sarah Eisenlord’s (University of Michigan) combined investigation of autecology and life history traits to enhance understanding of biogeographic patterns, and Eoin Brodie’s (Lawrence Berkeley National Laboratory) efforts to use trait-based predictions of actinobacteria abundances to describe microbial responses to long-term moisture conditions. Joe Zhou (University of Oklahoma) discussed his work using functional profiles from Geochip on soils sampled from complex field experiments, and explained how this approach can tackle intriguing questions related to how microbial species interact *in situ*. Discussion of the microbial *interactome* was particularly timely after the issues Don Klein (Colorado State University) raised regarding how interacting microbial communities are conceptualized and defined.

### Sequencing tradeoffs: how deep should we dig?

The tradeoffs between deep sequencing of a limited sample set and shallow sequencing of biological replicates emerged as a key dilemma facing experimentalists and has important ramifications for bioinformaticians. The greater data available from a deeply sequenced sample allows for better estimates of alpha diversity, better characterization of rare taxa, and the exciting potential for reconstructing whole genomes of unknown organisms. In addition, Yuzhen Ye (Indiana University) and Adina Howe (Michigan State University) noted that high-quality gene assembly and prediction rely on the high genomic coverage provided by deep sequencing. Conversely, Noah Fierer (University of Colorado, Boulder) and Konopka advocated that shallower sequencing might lower per-sample costs enough to make replicated experimental designs—critical for sound statistical inference—attainable [5]. Data from more samples, rather than more data per sample, may also make it easier to detect important patterns and the mechanisms responsible for them and to identify the function of unknown genes. Ultimately, the required magnitude of the sequencing effort depends on the question being asked; sequencing depth is an integral component of designing experiments that will generate interpretable data.

Even relatively shallow next-generation sequencing produces incredibly large volumes of short sequence fragments that must be assembled in order to determine what genes are present (*gene calling*). New or modified informatics tools will be required to handle such large datasets, and several creative strategies were presented. For example, Howe and Alice McHardy (Max-Planck Institute for Informatics) described new approaches to binning data prior to assembly, which should compress data without sacrificing information. Trevor Charles (University of Waterloo) described his approach to characterizing the function of genes before determining their sequence. Protein-based assembly was offered as another good way to handle the data deluge; Nikos Kyrpides (DOE Joint Genome Institute) described how such an informatics technique, called metafolding, can reduce dataset size without loss of functional information.

### Embedding metadata

Several speakers asserted that the paucity of standardized metadata limits how metagenomic sequence data can be used. Metadata—any information that accompanies sequence data, from site characteristics to edaphic measurements to methods descriptions—is critical for performing meaningful statistical analyses and providing context for interpretation of any sequencing project. Dawn Field (Centre for Ecology and Hydrology) summarized the efforts of the Genomic Standards Consortium (GSC), whose mission is to build a large, balanced community to solve problems related to metadata, and to define minimum standards for metadata [6]. Kyrpides described one tool for metadata curation, the Genomes OnLine Database [7], and Folker Meyer (Argonne National Laboratory) described how metadata entry would be incorporated in the latest version of the MG-RAST online tool for processing and analysis of sequence data [8]. Kevin Keegan (Argonne National Laboratory) used soils collected from throughout the US as a test case to demonstrate how extensive metadata can add a level of sophistication to the statistical tools available for interrogating metagenomic datasets. Another example of the utility of good metadata came from David Myrold (Oregon State University), who explained that the ideal outcome of his plans for an ambitious multi-omics project will be to use metadata to fit multi-omics data back into soil carbon models. As Field observed, community-accepted standards for metadata will provide the keys to unlocking new knowledge from metagenomic sequence data.

### Navigating the data bonanza

A network approach to data analysis is valuable for visualizing sequence data and for putting it in an interpretable ecological context. Computational scientists, including Ye, Howe, and McHardy, used network analysis to process sequence data prior to assembly. Likewise, Emmanuel Prestat (Université de Lyon) explained his application of similarity graphs and a Markov clustering algorithm to identify unknown sequences. Ecologists also employed network analysis to visualize interactions between genes and organisms. For example, Peter Larsen (Argonne National Laboratory) used expression modeling network analysis of mycorrhiza to understand phenotypes of fungi and trees given certain environmental conditions (elevated [CO_2_] and [O_3_]). The network-facilitated approach to data interpretation was also discussed in talks by Fierer and Zhou. Ashley Shade (Yale University) further extended the application, proposing that network analysis could be utilized with a series of community observations in space or time with analysis of their interactions to determine whether there is a community of organisms interacting with one another or a consortium of organisms that co-occur but do not interact.

Many speakers remarked that data production is outpacing computation capacity, which Field optimistically termed the *data bonanza*. Web-based platforms are needed to store and organize metagenomes to enable data exploration by investigators and relieve their computational burden. Such tools are becoming available, including MG-RAST (Argonne National Laboratory) and IMG/M (Joint Genome Institute). Furthermore, M5, a joint JGI and ANL pilot project, is tackling the big data problem by building a roadmap for scalable and sustainable computing metainfrastructure for the metagenomics community. Chris Henry (Argonne National Laboratory) presented a glimpse of the potential for sequencing projects to extend our knowledge of microbial systems with his innovative metabolic modeling of whole soil communities. Looking more broadly, Jim Tiedje (Michigan State University) reflected that this new field is in the groundwork phase, and that patience is required to move beyond rapid advances in sequencing to a computation-focused period. Only then can we expect a time of intense experimental work that will ultimately lead to a path of ecologically important discovery. Elizabeth Glass (Argonne National Laboratory) reinforced this view by emphasizing that metagenomics research must be based on an iterative process between biological inquiry and bioinformatics tools.

The clear message from this workshop was that carefully designed experiments are critical for harnessing the power of new sequencing platforms. This was particularly evident in the session *multi-omics challenges*, which featured speakers who are taking on the challenge of integrating metagenomics with metatranscriptomics, metaproteomics, and more. Kostas Konstantinidis (Georgia Institute of Technology) concluded that a clearly stated hypothesis is necessary for developing a systems perspective in soil microbial ecology. Clearly, it will take effort on all fronts (conceptual, experimental, computational) to fulfill the promise of a revolution in soil microbiology.

## What Changed in a Year

Soil metagenomics is gaining traction. Democratization of sequencing via cost-reducing innovations in sequencing technology will bring large-scale sequencing projects to more labs. As the same time, cloud computing is expanding the possibilities for handling the big data generated by such projects. With the metadata standards established by the GSC to tie it all together, we can expect soil metagenomics to mature from a boutique approach used by a handful of specialists to a sophisticated, highly interdisciplinary subfield. However, we must take care to “get it right” at this early stage so that the field can grow into a healthy, sustaining branch of science.

## Conclusions

Dionysios Antonopoulos (Argonne National Laboratory) wrapped up the workshop by identifying five key issues that emerged during the course of the 2010 workshop and need to be addressed if soil metagenomics studies are to produce ecologically meaningful results [6]. *Visualization for ever-larger datasets* ***–*** the sheer size and complexity of data generated by continually updated sequencing technology necessitates new, creative data visualization approaches that will allow experimentalists to interpret results in the context of well formulated hypotheses [3]. *Sample replication* – ever-increasing sequence capabilities and computational capacity will allow the field to mature beyond the point of single samples; robust, fully replicated designs must become the standard [7]. *Spatial structure and scaling issues* – it is clear that spatial structure produces patterns, but more work is required to determine how 2- and 3-D spatial structures drive function of microbial assemblages. Furthermore, we will have to solve the problem of how to conceptualize emergent properties from one level and translate them to the next [8]. *Sample economy* – extensive physical and chemical characterization required to implement GSC-mandated standards will consume material, especially low-volume samples that could help address Issue 3. (5) *Grounding in theory* – soon, data will no longer limit what can be learned about soil microbial communities. Instead, the ingenuity of research questions will determine how fast we advance. Careful experimental design grounded in theory developed in ecology and soil science must be employed to identify relationships that transcend individual sample sets and reveal ecological generalities. A key challenge for soil metagenomics is how to apply sequence data to questions in ecosystem ecology spanning from soil microsites to whole ecosystems. The 2^nd^ Annual Argonne Soils Workshop provided a platform for interdisciplinary discussion that helped define the goals for the next phase of ecologically meaningful soil metagenomics research.
